# Prevalence of Gastroesophageal Disease and Associated Risk Factors Among the Population in Al-Qunfudah

**DOI:** 10.7759/cureus.20325

**Published:** 2021-12-10

**Authors:** Mosad M Odah, Ashraf A Ewis, Awad A Alessi, Turki M Alhasani, Ali A Alghanmi, Abdulrahman A Almarhabi, Ibrahim M Almuashi, Ali A Almathami, Hassan O Alfakieh, Fuad M Alkudaysi, Ibrahim A Alnashri, Hassan I Alnashri, Mohammed A Awad, Mohammed S Alammari, Adnan A Alessa

**Affiliations:** 1 Biochemistry, Umm Al-Qura University, Al-Qunfudah, SAU; 2 Public Health, Umm Al-Qura University, Al-Qunfudah, SAU; 3 Medicine and Surgery, Umm Al-Qura University, Al-Qunfudah, SAU

**Keywords:** saudi arabia, al-qunfudah, risk factors, prevalence, gerd

## Abstract

Background and objective

Gastroesophageal reflux disease (GERD) is one of the most common gastrointestinal diseases worldwide. It causes an unpleasant effect on patients' lives and may lead to serious complications resulting in a significant burden on healthcare systems. Despite being a common gastrointestinal disease, very few studies have been conducted on the condition in Saudi Arabia; and there has never been a study to estimate the prevalence of GERD in the Al-Qunfudah Governorate. In light of this, we conducted this study with an aim to assess the prevalence of GERD and its associated risk factors among the Al-Qunfudah population.

Methods

A cross-sectional study was conducted in the Al-Qunfudah Governorate by using an online self-administrated questionnaire that was shared through social media during the first week of January 2021. The questionnaire consisted of a general section on sociodemographic data and a section on the diagnosis of GERD based on the validated gastroesophageal reflux disease questionnaire (GERD-Q). A total of 1,180 eligible participants responded to the questionnaire.

Results

Nearly one-third (32.9%) of the study participants had GERD based on their reported symptoms and calculated scores (GERD-Q score ≥8). About 14.8% of the participants (175/1,180) reported that they had already been diagnosed with GERD before their participation in our survey. Regarding risk factors of GERD, about 35% reported experiencing psychological stress, 28.3% had a family member diagnosed with GERD, and 18.1% were smokers; 49.4% of the participants had their symptoms aggravated by consuming fatty or fried food and 46.7% by spicy food. One of the factors that helped to relieve GERD symptoms was avoiding symptom-aggravating food, as reported by more than half (50.7%) of the participants.

Conclusion

The prevalence of GERD in the Al-Qunfudah population is high as the condition has affected one-third of the adult population. Our study confirms that male gender, age of 30 years or above, being overweight or obese, being married, smoking habit, use of non-steroidal anti-inflammatory drugs (NSAIDs), having psychological stress, being asthmatic, or having a family history of GERD are factors that significantly increase the likelihood of developing GERD. The reported risk factors include experiencing psychological stress, a family history of GERD, high BMI, and smoking.

## Introduction

Gastroesophageal reflux disease (GERD) is a widely prevalent gastrointestinal disease worldwide [1]. It occurs when there is an inappropriate relaxation of the lower esophageal sphincter (LES) resulting in the backflow of the gastroduodenal contents into the mucosa of the esophagus. When this happens for extended periods of time, it leads to symptoms including heartburn, acid regurgitation, and chest or epigastric discomfort [1,2].

Many risk factors contribute to the development of GERD, including obesity, dietary factors such as the consumption of caffeine, spicy food, and carbonated beverages, smoking, non-steroidal anti-inflammatory drugs (NSAIDs) use, psychological stress, asthma, family history of heartburn or GERD, older age, hiatus hernia, and alcohol consumption; however, most of these risk factors are modifiable [2].

GERD decreases the quality of life, affecting patients physically and socially, resulting in a negative impact on daily activities, sleep, and work productivity. GERD can also lead to serious complications such as erosive esophagitis, Barrett's esophagus, and esophageal adenocarcinoma, which eventually causes a significant burden on healthcare systems [3,4].

Globally, there is significant variation in the prevalence of GERD among different populations. A systemic review study showed a prevalence of GERD between 8.7-33.1% in the Middle East [3], and a cross-sectional study in Saudi Arabia has shown it to be 28.7% in the country [5]. Recent studies have shown high and varying prevalence rates of GERD (Gerd-Q score ≥8) among adults in different cities of Saudi Arabia - Makkah: 23.5% [6], Jazan: 32.2% [7], Riyadh: 45.4% [8], and Arar: 61.8% [9]. Despite being a common gastrointestinal disease, very few studies have been conducted on GERD in Saudi Arabia, and no study has been done so far to estimate the prevalence of GERD in the Al-Qunfudah Governorate. Hence, the aim of this study was to examine the prevalence of GERD and determine its risk factors among the Al-Qunfudah population.

## Materials and methods

A cross-sectional study was conducted among the population of Al-Qunfudah Governorate to assess the prevalence of GERD and its associated risk factors. Al-Qunfudah Governorate is located in the southern part of Makkah Al-Mukarramah Province, Saudi Arabia, and it is one of the coastal governorates in the region. The governorate encompasses an area of about 5,195 km^2^ with a total population of 194,811 [10]. The calculated required sample size for the study was 385 with a confidence level (CL) of 95% and confidence interval (CI) of 5%. Data were collected during the first week (1st-7th) of January 2021. An online self-administrated questionnaire was shared through social media apps (WhatsApp, Snapchat, and Telegram), targeting the general population who live in the Al-Qunfudah Governorate and were aged 18 years or above. About 1,180 eligible respondents answered the questionnaire and participated in the study.

The questionnaire included questions designed to attain the study objectives. Items in the questionnaire were categorized into four parts. The first part included questions about the sociodemographic data of the participants such as age, sex, educational level, and marital status. The second part entailed the GERD-Q, which is the validated diagnostic tool for GERD. The third part of the questionnaire included queries meant for assessing the prevalence of the risk factors for GERD, e.g., obesity, smoking, use of NSAIDs, psychological stress, asthma, family history of GERD, alcohol intake, and hiatus hernia. The fourth part inquired about the aggravating and relieving factors with respect to the GERD symptoms. Aggravating factors comprised aspects such as drinking tea, coffee, or soft drinks, and eating spicy, acidic, fatty, or fried food, and relieving factors consisted of items such as taking medication, avoiding symptom-aggravating food, or changing the sleeping position.

The GERD-Q is a tool that helps to diagnose GERD, and it consists of six questions including four positive indicators (regurgitation, heartburn, sleep difficulties due to heartburn and/or regurgitation, and the use of medications for GERD symptoms without a prescription), and two negative indicators (nausea and epigastric pain) during the previous seven days. Each question about positive indicators is rated on a 4-point Likert scale (0=none, 1=one day, 2=two to three days, and 3=four to seven days), and a reversed Likert scale (3=none) for two negative indicators of GERD, giving a range from 0-18 of the total GERD-Q score; those who received scores of 8 or above are more likely to have GERD. It has a sensitivity of 65% and a specificity of 71% for GERD diagnosis [11]. Since there was no validated Arabic GERD-Q available, we translated the questionnaire and revised it with the help of experts and professionals, and tested the final version on 25 subjects in a pilot study before sharing it with the target population.

Data were collected, cleaned up, entered into, coded, and analyzed using the SPSS Statistics software version 23 (IBM, Armonk, NY). Categorical variables were presented as numbers and percentages. Crude odds ratios (OR) and their 95% CI were calculated. Significant factors associated with GERD on bivariate analysis were entered into multivariate logistic regression models using the stepwise forward Wald method to detect the independent predictor of risk to develop GERD. A p-value ≤0.05 was considered statistically significant.

## Results

A total of 1,180 people participated in the study. The majority of them were males (63.5%), while females constituted 36.5%. The mean age of the participants was 31.7 ±10.7 years, and about 53.7% of them were married and 43.6% were single. More than two-thirds (67.9%) of the respondents had a bachelor’s degree. Regarding the BMI, nearly half of the participants (50.6%) were overweight or obese (BMI ≥25 kg/m^2^). With regard to the prevalence of the risk factors for GERD, about 18.1% were smokers, 13.6% were asthmatic, 35% were experiencing psychological stress, and 28.3% had a family member diagnosed with GERD. About 14.8% of the participants (175/1,180) reported that they already had GERD at the time of our survey, and 75.4% of them had been diagnosed with GERD at the clinic by physicians (Table 1).

**Table 1:** Sociodemographic data of the participants in the GERD study among Al-Qunfudah population, Saudi Arabia BMI: body mass index; NSAIDs: non-steroidal anti-inflammatory drugs; SD: standard deviation

Variables			Values (n=1,180)
Age (years)	Mean ±SD (range)		31.7 ±10.7 (16-94)
	<30 years		572 (48.5%)
	≥30 years		608 (51.5%)
Sex, n (%)	Male		749 (63.5%)
	Female		431 (36.5%)
Marital status, n (%)		Single	514 (43.6%)
		Married	634 (53.7%)
		Divorced	20 (1.7%)
		Widowed	12 (1.0%)
Educational level, n (%)		High school degree or less	196 (16.6%)
		Diploma	131 (11.1%)
		Bachelor’s degree	801 (67.9%)
		Master’s degree and above	52 (4.4%)
Weight (kg)	Mean ±SD (range)		71.4 ±17.2 (40-133)
Height (cm)	Mean ±SD (range)		165.1 ±8.7 (149-190)
BMI (kg/m^2^)	Mean ±SD (Range)		26.1 ±5.5 (16.7-50.8)
Weight groups, n (%)		Underweight (<18.5 kg/m^2^)	64 (5.4%)
		Healthy weight (18.5-24.9 kg/m^2^)	519 (44.0%)
		Overweight (25-29.9 kg/m^2^)	337 (28.6%)
		Obese I (30-34.9 kg/m^2^)	167 (14.2%)
		Obese II (35-39.9 kg/m^2^)	76 (6.4%)
		Obese III (≥40 kg/m^2^)	17 (1.4%)
Do you have information about gastroesophageal reflux disease? N (%)		Yes	757 (64.2%)
		No	423 (35.8%)
Have you been diagnosed with gastroesophageal reflux disease? N (%)		Yes	175 (14.8%)
		No	1,005 (85.2%)
How were you diagnosed? N (%)		At the clinic by a doctor	132 (75.4%)
		By esophagogastroduodenoscopy	40 (22.9%)
		By ambulatory 24-hour pH monitoring	3 (1.7%)
Smoking, n (%)		Current smoker	214 (18.1%)
		Ex-smoker	66 (5.6%)
		Non-smoker	900 (76.3%)
Alcohol, n (%)		Yes	21 (1.8%)
		No	1,159 (98.2%)
NSAIDs use, n (%)		Yes	150 (12.7%)
		No	1030 (87.3%)
Asthma, n (%)		Yes	160 (13.6%)
		No	1,020 (86.4%)
Psychological stress, n (%)		Yes	413 (35.0%)
		No	767 (65.0%)
Family member diagnosed with the gastroesophageal disease, n (%)		Yes	334 (28.3%)
		No	846 (71.7%)
Diagnosed with hiatus hernia, n (%)		Yes	28 (2.4%)
		No	1,152 (97.6%)

Regarding the prevalence of the main symptoms of GERD, our results showed that about 49.1% of the participants reported suffering from heartburn and 47.5% reported having regurgitation (Table 2).

**Table 2:** Frequency of GERD symptoms among participants from Al-Qunfudah population, Saudi Arabia GERD: gastroesophageal reflux disease

Reported symptoms of GERD	Never, n (%)	One day/week, n (%)	2-3 days/week, n (%)	4-7 days/week, n (%)
Heartburn	601 (50.9%)	299 (25.3%)	186 (15.8%)	94 (8.0%)
Regurgitation	620 (52.5%)	343 (29.1%)	148 (12.5%)	69 (5.8%)
Pain in the middle of the upper stomach area	591 (50.1%)	319 (27.0%)	189 (16.0%)	81 (6.9%)
Nausea	764 (64.7%)	214 (18.1%)	138 (11.7%)	64 (5.4%)
Trouble getting a good night's sleep because of heartburn or regurgitation	756 (64.1%)	246 (20.8%)	129 (10.9%)	49 (4.2%)
Additional medication	914 (77.5%)	123 (10.4%)	84 (7.1%)	59 (5.0%)

With regard to the prevalence of GERD among respondents, nearly one-third of them (32.9%) had GERD based on their calculated scores (GERD-Q score ≥8) (Table 3).

**Table 3:** GERD score of the studied individuals from Al-Qunfudah, Saudi Arabia GERD: gastroesophageal reflux disease; SD: standard deviation

Variables		Values (n=1,180)
GERD score	Mean ±SD (range)	7.1 ±2.3 (0-16)
Likelihood of GERD	0% likelihood of GERD	8 (0.7%)
	50% likelihood of GERD	784 (66.4%)
	79% likelihood of GERD	273 (23.1%)
	89% likelihood of GERD	115 (9.7%)
GERD	Low likelihood of GERD (≤50%)	792 (67.1%)
	High likelihood of GERD (≥79%)	388 (32.9%)

As for the aggravating symptoms of GERD, about 49.4% of the participants reported that their symptoms are aggravated by consuming fatty or fried food, 46.7% by consuming spicy food, and 34.4% by drinking coffee (Table 4).

**Table 4:** Factors that aggravate GERD symptoms among Al-Qunfudah population, Saudi Arabia GERD: gastroesophageal reflux disease

Aggravating factor	Yes		No	
	N	%	N	%
Drinking coffee	406	34.4%	774	65.6%
Drinking tea	210	17.8%	970	82.2%
Eating spicy food	551	46.7%	629	53.3%
Eating acidic food such as orange and lemon	274	23.2%	906	76.8%
Eating fatty or fried food	583	49.4%	597	50.6%
Eating tomato products such as sauce and ketchup	322	27.3%	858	72.7%
Consuming soft drinks	218	18.5%	962	81.5%
Eating large amounts of food	316	26.8%	864	73.2%
Eating meals late at night	339	28.7%	841	71.3%
None	225	19.1%	955	80.9%

One of the factors that helped to relieve GERD symptoms among the participants was avoiding symptom-aggravating food, as reported by more than half of the participants (50.7%), followed by taking medication (31.6%) (Table 5).

**Table 5:** Factors that help to relieve GERD symptoms in participants from Al-Qunfudah, Saudi Arabia GERD: gastroesophageal reflux disease

Relieving factor	Yes		No	
	N	%	N	%
Taking medication	373	31.6%	807	68.4%
Avoiding symptom-aggravating food	598	50.7%	582	49.3%
Changing sleep position	207	17.5%	973	82.5%
None	342	29.0%	838	71.0%

Univariate and multivariate binary logistic regression analyses of the factors associated with a high likelihood of GERD are presented in Table 6. The findings showed that male gender, age of 30 years or above, being overweight or obese, being married, smoking habit, use of NSAIDs, having psychological stress, being asthmatic, or having a family history of GERD are factors that significantly increase the likelihood of developing GERD.

**Table 6:** Univariate and multivariate binary logistic regression analysis of factors associated with a high likelihood of GERD among Al-Qunfudah population, Saudi Arabia *Statistically significant GERD: gastroesophageal reflux disease; OR: odds ratio; CI: confidence interval; BMI: body mass index; NSAIDs: non-steroidal anti-inflammatory drugs

Variables		Crude OR (95% CI)	P-value	Adjusted OR (95% CI)	P-value
Sex	Male	1.96 (1.50-2.55)	<0.001*	2.25 (1.69-3.01)	<0.001*
	Female	1 (reference)		1 (reference)	
Age	≥30 years	2.09 (1.63-2.69)	<0.001*	1.57 (1.09-2.26)	0.016*
	<30 years	1 (reference)		1 (reference)	
BMI	Underweight	1.24 (0.70-2.19)	0.464	1.56 (0.86-2.84)	0.145
	Overweight	1.94 (1.44-2.60)	<0.001*	1.55 (1.13-2.13)	0.007*
	Obese	1.92 (1.40-2.64)	<0.001*	1.49 (1.06-2.10)	0.023*
	Healthy weight	1 (reference)		1 (reference)	
Marital status	Married	2.12 (1.65-2.73)	<0.001*	1.53 (1.06-2.21)	0.022*
	Unmarried	1 (reference)		1 (reference)	
Education	High school	1.69 (0.83-3.44)	0.146	2.75 (1.29-5.87)	0.009*
	Intermediate	2.19 (1.05-4.57)	0.036*	3.96 (1.81-8.69)	0.001*
	University	1.58 (0.82-3.07)	0.173	2.70 (1.33-5.47)	0.006*
	Post-graduate	1 (reference)		1 (reference)	
Smoking		1.55 (1.14-2.10)	0.005*		
NSAIDs use		1.62 (1.14-2.30)	0.007*	1.54 (1.05-2.26)	0.026*
Asthma		1.62 (1.15-2.27)	0.006*		
Psychological stress		1.82 (1.42-2.34)	<0.001*	2.03 (1.54-2.68)	<0.001*
Family history of gastroesophageal disease		1.77 (1.36-2.30)	<0.001*	1.73 (1.30-2.30)	<0.001*
Alcohol use		1.88 (0.79-4.46)	0.153		
Hernia		1.80 (0.85-3.81)	0.127		

## Discussion

This cross-sectional study was conducted to estimate the prevalence of GERD and associated risk factors among the population of Al-Qunfudah Governorate. It showed a high prevalence of 32.9% in comparison to Makkah (western region, 23.5%) [6] and was almost equal to that of Jazan (southwestern region, 32.2%) [7]. However, the prevalence was low compared to Riyadh (central region, 45.4%) [8], and Arar (northern region, 61.8%) [9]. The prevalence of GERD in the whole of Saudi Arabia is reported to be 28.7% [5]. In a systematic review including only seven studies from various parts of the Middle East (five in Iran, one in Turkey, and one in Israel) the authors found a prevalence range of 8.7-33.1%. It was higher than the prevalence of GERD in North America (18.1-27.8%), Europe (8.8-25.9%), East Asia (2.5-7.8%), Australia (11.6%), and South America (23.0%) [12].

In this study, the common positive symptoms reported by participants were heartburn (49.1%) and regurgitation (47.5%), which is in line with the findings of previous studies [1,2,5,13]. The common negative symptoms were epigastric pain (49.9%) and nausea (35.3%).

A meta-analysis study has shown the prevalence of GERD to be higher in subjects aged ≥50 years (OR: 1.32; 95% CI: 1.12-1.54) [14]. We also found that individuals aged ≥30 years have a 1.57 times higher risk for GERD compared to the younger population (adjusted OR: 1.57; 95% CI: 1.09-2.26). Another systematic study found that GERD was significantly more common in men than in women [15], suggesting that this could be related to the impact of the sex hormone estrogen in females. This study demonstrated a double risk for the male gender to have GERD in contrast to females (adjusted OR: 2.25; 95% CI: 1.69-3.01). However, some other studies have shown no association between gender and GERD [5,8,9].

The American college of gastroenterology (ACG) has determined obesity to be one of the major risk factors for GERD and recommends losing weight for those who are overweight [16]. Many studies have referred to an association between higher BMI levels and GERD [17,18,19]. There was a strong association in our study between higher BMI levels and the likelihood of GERD (Table 6).

A systemic review article that studied the relationship between GERD and psychological comorbidities has reported that they are common among GERD patients [20]. In addition, they may lead to failure of response to treatment. In this study, we found a significant relationship between psychological stress and a high likelihood of GERD (adjusted OR: 2.03; 95% CI: 1.54-2.68). Also, GERD symptoms are significantly related to family history of the disease; a study from Shiraz city (Iran) involving 1,956 subjects has revealed that among 72% of the participants with GERD symptoms, at least one member of the family had a history of GERD (p<0.001) [21]. Our results showed that individuals with a family history of GERD had 1.73 times the risk for GERD compared to those without (adjusted OR: 1.73; 95% CI: 1.30-2.30) (p<0.001).

Smoking and the use of NSAIDs are known risk factors for GERD [22-24]. In our cohort, 18.1% were smokers and 5.6% were ex-smokers while 12.7% were using NSAIDs. These aspects were highly associated with GERD (Table 6). In a systemic review involving 28 studies, the average prevalence of GERD symptoms in asthma patients was 59.2%, whereas it was 38.1% in controls. In comparison, the average prevalence of asthma in individuals with GERD was 4.6%, whereas it was 3.9% in controls [25]. The prevalence of asthma in this study was 13.6% and there was a high likelihood of GERD in patients with asthma (OR: 1.62; 95% CI: 1.15-2.27) (p=0.006).

A recent systematic review has concluded that dietary habits play a major role in exacerbating GERD symptoms. Spicy food, eating citrus fruits with meals, fried foods, greasy foods, eating snacks at night, frequently skipping breakfast, eating quickly, eating hot food, and overeating are all positively correlated with GERD. In contrast, vegetarian diets, fruits, vegetables, vitamins, fiber, and proper physical exercise were negatively correlated with GERD [26]. Among the participants in our study, fatty, fried, and spicy foods were the major dietary factors that aggravated the symptoms of GERD: 49.4% reported symptom aggravation by consuming fatty and fried food, 46.7% by consuming spicy food, and 27.3% by eating tomato products such as tomato sauce and ketchup. Drinking coffee had a higher association (34.4%) compared to tea drinking (17.8%) with aggravating symptoms of GERD. Furthermore, eating acidic food such as orange and lemon was associated with aggravated symptoms in 23.2% due to the increased acid levels in the stomach. Eating habits have a significant impact on exacerbating the symptoms of GERD; 28.7% of the participants reported that their symptoms worsened by eating meals late at night and 26.8% by eating large amounts of food (Table 4). Factors that help to relieve GERD symptoms were avoiding symptom-aggravating food (50.7%) and taking medications (31.6%) (Table 5).

Many studies have reported that alcohol consumption is a risk factor for GERD [2,17,26]. In our study, we could not evaluate this aspect as alcohol consumption is prohibited in Saudi Arabia. Also, we did not observe any association between hiatus hernia and GERD in this study.

This study has some limitations that should be addressed. The cross-sectional design of the study restricted the ability to differentiate newly diagnosed cases from long-term sufferers without an established diagnosis. Moreover, because of the lockdown imposed due to the coronavirus disease 2019 (COVID-19) pandemic, which coincided with the data collection period in Saudi Arabia, we had to exclusively depend on the online survey format for data collection, and this could have introduced a non-response bias that could undermine the generalization of the study findings since the non-respondents might carry different characteristics compared to the respondents. To mitigate the impact of this bias, we tried our best to give the maximum number of people among the target population access to the GERD questionnaire by forwarding the link of the online Google Form via different social networks. Additionally, we extended the data collection period by two weeks to give as many respondents as possible the opportunity to participate in the survey. Also, the association between stress and GERD needs to be investigated further in future studies.

## Conclusions

Based on our findings, the prevalence of GERD among the population in Al-Qunfudah is high and accounts for one-third of the adult population. Our study confirms that male gender, age of 30 years or above, being overweight or obese, being married, smoking habit, use of NSAIDs, having psychological stress, being asthmatic, or having a family history of GERD are factors that significantly increase the likelihood of developing GERD. The reported risk factors for GERD include experiencing psychological stress, a family history of GERD, high BMI, and smoking. Consuming fatty, fried, and spicy food plays a role in aggravating symptoms of GERD. Avoiding food and drinks that trigger symptoms, quitting smoking, and maintaining a healthy weight can aid in reducing the symptoms of GERD. We recommend raising awareness about GERD and its associated risk factors through public education and health-related programs to improve the quality of life and prevent further complications.
